# Character Strengths as Predictors of Mental Health and Well-Being During the COVID-19 Pandemic: A 13-Month Longitudinal Study

**DOI:** 10.3390/ijerph23010074

**Published:** 2026-01-04

**Authors:** María Luisa Martínez-Martí, Cecilia I. Theirs, David Pascual, Sergio Villar

**Affiliations:** 1 Facultad HM de Ciencias de la Salud, Universidad Camilo José Cela, C/Castillo de Alarcón, 49. Urb, Villafranca del Castillo, 28692 Madrid, Spain; citheirs@ucjc.edu (C.I.T.); sergio.villar@ucjc.edu (S.V.); 2 Facultad de Educación, Universidad Camilo José Cela, C/Castillo de Alarcón, 49. Urb, Villafranca del Castillo, 28692 Madrid, Spain; dpascual@ucjc.edu

**Keywords:** character strengths, mental health, well-being, positive affect, negative affect, life satisfaction, posttraumatic growth, COVID-19 pandemic, resilience, longitudinal study, mediation model, SEM

## Abstract

The COVID-19 pandemic has posed significant challenges to mental health worldwide, raising the need to identify stable psychological resources that promote sustainable well-being. This longitudinal study examined whether character strengths predict well-being, post-traumatic growth, and mental health over a 13-month period. Participants (N = 146) completed online measures of character strengths, mental health, life satisfaction, affect, and post-traumatic growth at two time points. First, we tested whether a single general factor of character predicted later mental health and whether life satisfaction, affect, and post-traumatic growth mediated this relationship. Then, we repeated this model but with five different character strengths factors as predictors. Results showed that character predicted all mediators and mental health over time, but only the affective components of well-being mediated the relationship between character and mental health, especially positive affect. When looking at the five character strengths factors, although the majority predicted higher well-being and better mental health over time, goodness and interpersonal and fortitude strengths yielded the strongest effects. These findings suggest that character strengths contribute to sustainable well-being by fostering affective resilience in the face of adversity, aligning with the goals of Sustainable Development Goal 3 (Good Health and Well-being).

## 1. Introduction

Well-being plays a central role in human functioning and is a major focus of contemporary psychological research. Its relevance at the societal level is reflected in its designation as a priority within the 2030 Agenda for Sustainable Development, particularly in Sustainable Development Goal 3 (Good Health and Well-being). This global priority highlights the need to understand which psychological resources help individuals maintain well-being across time and contexts—an increasingly urgent task considering today’s complex social and environmental challenges.

The scale of this challenge becomes evident when considering global patterns in mental health. The latest World Mental Health Report [1] highlights that one in eight individuals worldwide suffers from a mental disorder and identifies large-scale stressors—such as the COVID-19 pandemic—as key obstacles to mental health. Global data show that rates of common mental disorders, including depression and anxiety, increased by 25% during the first year of the pandemic, adding to the nearly one billion people already experiencing a mental health condition. Yet, despite this widespread psychological impact, some individuals reported positive psychological changes, such as greater appreciation of life or increased personal strength—hallmarks of post-traumatic growth (PTG) [2]. These divergent responses underscore the importance of identifying personal characteristics that promote adaptive functioning under adversity.

Within this context, an essential question emerges: what psychological factors can promote sustainable well-being and protect individuals from developing mental health problems, particularly during periods of prolonged stress? If the goal is to foster well-being that endures over time and across situations, then attention must turn to relatively stable psychological characteristics, including personality traits. Among these, character strengths have received increasing empirical attention as potential sources of well-being and resilience (e.g., [3,4]). Although a substantial body of research links character strengths to a range of well-being outcomes, their role in supporting sustainable well-being and mental health in challenging contexts—such as the COVID-19 pandemic—remains less understood.

### 1.1. Character Strengths, Well-Being and Post-Traumatic Growth

Character strengths—defined by Peterson and Seligman [5] as positive, morally valued, and relatively stable personality traits—represent a promising psychological resource for sustaining well-being under adversity. Drawing on an extensive review of philosophical and religious traditions, these authors proposed a classification of six universal virtues expressed through 24 character strengths, which together outline the pathways through which individuals enact moral excellence in daily life (Table 1). This framework positions character strengths as core components of positive human functioning and as stable individual capacities that can support adaptive responses to life challenges.

Examining the contribution of character strengths to psychological functioning involves considering their links to different forms of well-being. In the literature, these forms are commonly grouped into two complementary dimensions. The hedonic dimension, reflected in subjective well-being (SWB), refers to the experience of positive emotions, low levels of negative emotions, and life satisfaction [6], capturing both the affective and cognitive components of subjective evaluation. The eudaimonic dimension, reflected in psychological well-being (PWB), emphasizes positive psychological functioning and represents a central component within broader conceptualizations of flourishing, incorporating elements such as meaning, autonomy, and personal growth [7]. Together, these dimensions provide a comprehensive framework for examining how stable individual traits contribute to mental health over time.

Beyond these two dimensions, post-traumatic growth (PTG) constitutes an additional construct of particular relevance during adversity. PTG refers to perceived positive psychological changes that arise from the struggle with major stressors, including transformations in self-perception, interpersonal relationships, and life philosophy [8]. These changes reflect a process of cognitive reorganization through which individuals reorder priorities, reconstruct meaning, and develop a revised understanding of themselves and the world. Although conceptually distinct from well-being, PTG shares with eudaimonic perspectives an emphasis on meaning, growth, and positive transformation [2]. In this sense, PTG can be considered a specific form of positive adaptation that, while not equivalent to eudaimonic well-being, is deeply connected to its core processes of meaning and growth.

A growing body of empirical research demonstrates that character strengths are meaningfully connected to these different components of well-being and mental health. Strengths such as hope, gratitude, love, curiosity, and zest have been consistently associated with higher SWB, lower psychological distress, and better overall mental health [3,4]. Other work shows that character strengths also support eudaimonic forms of adaptation, including PTG [2,8], suggesting that they facilitate deeper forms of personal development when individuals confront significant stress. Moreover, longitudinal studies conducted before and during the COVID-19 pandemic indicate that character strengths remain relatively stable over time [9,10,11], reinforcing their relevance as enduring resources for promoting sustainable well-being. This stability aligns with the aims of SDG 3, which emphasizes the importance of strengthening individual capacities that sustain health across diverse contexts and extended periods.

### 1.2. Character Strengths, Well-Being and Post-Traumatic Growth Under the COVID 19 Pandemic

The COVID-19 pandemic constituted an unprecedented global stressor, characterized by prolonged uncertainty, social isolation, and a long lockdown, which collectively led to significant declines in mental health across the general population. Research has consistently documented substantial increases in psychological distress during this period, including elevated symptoms of depression, anxiety, and stress (e.g., [12]), with longitudinal evidence indicating that these difficulties remained stable or even worsened over time (e.g., [13]). Despite this overall negative impact, several studies have also reported signs of well-being and resilience (e.g., [3,4]). In addition, accumulating evidence shows the presence of post-traumatic growth following the pandemic (e.g., [14,15]), a finding reinforced by recent systematic reviews that document pandemic-related PTG across diverse populations and its consistent association with resilience, optimism, social support, and cognitive reappraisal, as well as its co-occurrence with elevated post-traumatic stress symptoms [16]. This coexistence of distress and growth highlights the need to identify psychological resources that promote resilience and positive adaptation during sustained adversity.

Recent longitudinal studies show initial evidence of the association between character strengths and well-being (including PTG) and mental health (MH) in the context of the COVID-19 pandemic. For example, Martínez-Martí et al. [9] observed that, generally, character strengths predicted SWB and MH one month later during the COVID-19 pandemic lockdown in Spain, i.e., at the beginning of the pandemic. In that study, character strengths were grouped empirically into five factors (see Table 2).

All five character strengths factors predicted better MH, and—except strengths of restraint—higher positive affect. Furthermore, intellectual and interpersonal strengths predicted higher life satisfaction. Finally, fortitude strengths, interpersonal strengths, and strengths of restraint predicted lower negative affect. Overall, fortitude strengths yielded the highest correlations with MH and SWB.

Evidence from other longitudinal studies extends these findings. In an eight-month study conducted during the first year of the pandemic, Casali, Feraco, and Meneghetti [10] showed that character—operationalized as a single higher-order factor encompassing all 24 VIA strengths—had a direct protective effect on mental health and an indirect effect through post-traumatic growth (PTG), indicating that individuals with higher dispositional character were more likely to maintain better psychological functioning over time and to experience positive psychological changes despite ongoing stressors. In addition, their results revealed that among the six VIA virtues, transcendence uniquely predicted better mental health at Time 2, whereas humanity uniquely predicted PTG, highlighting the specific components of character most closely associated with adaptive adjustment during the pandemic. Complementing this, Gander and Wagner [11] examined strengths assessed up to 1.5 years before the onset of COVID-19 and found that several specific strengths—particularly zest, love, kindness, appreciation of beauty and excellence, gratitude, and spirituality—were positive predictors of PTG during the pandemic, suggesting that pre-existing dispositional strengths play a substantial role in shaping adaptive responses to collective adversity. Together, these longitudinal findings indicate that both broad character resources and specific strengths measured well before the crisis can support positive adjustment, offering initial evidence that dispositional character functions as an enduring psychological asset in contexts of prolonged disruption.

However, since the longitudinal studies that explore the relationship between character strengths, well-being, and MH in the context of the challenges we face today, such as pandemics, are still very scarce, more and longer longitudinal studies are needed. Moreover, these studies focus on either hedonic components of well-being or post-traumatic growth separately, offering a fragmented picture of how character strengths relate to well-being and mental health.

### 1.3. The Current Study

To fill these gaps, this study examines whether character strengths predict mental health over a longer period, in comparison with previous studies. Moreover, in an attempt to synthetize previous evidence relating character strengths and different components of well-being and mental health, we test a unified model in which we explore simultaneously different well-being routes through which character strengths might lead to better mental health. Specifically, we test whether character strengths lead to (1) higher positive affect, (2) lower negative affect, (3) higher life satisfaction (all components reflecting hedonic well-being), and (4) higher post-traumatic growth (reflecting positive psychological change following adversity), which in turn lead to better MH. Finally, since this study is an extension of a previous investigation [9] that explored the relationship between character strengths, MH and SWB, over a one-month period during the initial stages of the pandemic, we further explore which character strengths remain significant predictors of WB and MH in the long run. The inclusion of PTG, which has been consistently linked to better MH, together with the hedonic well-being indicators, strengthens the theoretical grounding of the model and help clarify the mechanisms linking strengths and MH.

To address these questions, we first estimated a structural equation model (SEM) with AMOS in which a latent character factor—comprising five empirically derived strength dimensions—served as the main predictor, with positive affect, negative affect, life satisfaction, and post-traumatic growth at T2 as mediators and MH at T2 as the outcome, controlling for baseline MH and sociodemographic variables. This model assessed whether a global character factor (as in Casali et al. [9]), predicts well-being and long-term MH, offering a broad and parsimonious view. In a second step, to obtain a more detailed picture, we conducted five parallel mediation models with IBM SPSS Statistics 29 (IBM, Armonk, NY, USA) and the PROCESS v4.2 macro (Cochrane, London, UK), each using one character strength factor as predictor and the same mediators and outcome. This complementary analysis allowed us to examine whether specific strength domains exert different effects.

We expect that the single character factor leads to (1) higher positive affect, (2) lower negative affect, (3) higher life satisfaction, and (4) higher post-traumatic growth, which in turn leads to better MH. Additionally, we expect the five character strengths factors to follow this general pattern but with some differences. Specifically, and following previous evidence (e.g., [9,10,11]), we expect that all five character strengths factors predict higher positive affect, which in turn leads to better MH, except strengths of restraint. Furthermore, we expect intellectual and interpersonal strengths to predict higher life satisfaction, which in turn leads to better MH and fortitude strengths, interpersonal strengths, and strengths of restraint to also predict lower negative affect, which in turn leads to better MH. As for the PTG route, we expect the character strengths factors of fortitude, goodness and intellect to be especially related to PTG, which in tun leads to better MH.

## 2. Materials and Methods

### 2.1. Participants

The sample comprised 146 participants (112 female, 34 male) with mean age M = 44.4 years (SD = 11, range 21–76). By the time of the first data collection (T1, from 21 March 2020 to 2 April 2020), all participants were residents in Spain. Most of them were Spanish (94.5%), followed by German (1.4%) and other nationalities that each represented 0.7% such as American, Italian, or Swiss. Regarding education, 50% of the sample had a university degree or diploma, 29.5% had completed postgraduate studies, 11% had a PhD, and 9.5% had graduated from secondary school.

### 2.2. Measures

The Spanish version of the Character Strengths Rating Form (CSRF; [17]), which has shown good psychometric properties [9], was used to assess the 24 character strengths. It has 24 items, and each item assesses one of the 24 character strengths with a 9-point Likert scale (from 1 = not like me at all through 9 = absolutely like me). Higher scores represent a higher endorsement of the strength. A sample item is “Spirituality: having coherent beliefs about the higher purpose and meaning of life.” We grouped the 24 character strengths as in the study by Martínez-Martí et al. [9]. In this previous study, a principal component analysis was conducted, finding that the 24 character strengths were grouped into five factors (Table 2). In the present study, the factor analysis revealed a highly similar structure (only four items, out of 24, presented higher loading in other factors). Considering that the indexes of internal consistency of the five factors were good, we decided to retain those five character strengths factors to be able to compare with the previous study [9]. In the current study, Cronbach’s alphas and Omega were, respectively, 0.75 and 0.75 (fortitude strengths), 0.82 and 83. (goodness strengths), 0.81 and 81. (intellectual strengths), 0.73 and 0.76 (interpersonal strengths), and 0.75 and 0.77 (strengths of restraint), showing acceptable-good internal consistency. Mean scores were used for subsequent analyses.

The Spanish version [18] of the 12-item General Health Questionnaire (GHQ-12; [19]) was used to assess mental health, including social functioning, loss of confidence, depression, or anxiety. Participants rated the items considering their experience during the last week on a 4-point Likert-type scale (0 to 3). Higher scores indicate worse mental health. In the present study, Cronbach’s alphas and Omega were both 0.83 (T1) and 0.90 (T2). Sum scores were used for subsequent analyses.

The Spanish version [20] of the 5-item Satisfaction with Life Scale (SWLS; [21]) was used to measure life satisfaction in a 7-point Likert scale (from 1 = strongly disagree to 7 = strongly agree). Higher scores reflect higher life satisfaction. A sample is “I am satisfied with my life”. In the present, Cronbach’s alphas and Omega were both 0.87. Sum scores were used for subsequent analyses.

The Spanish version [22] of the Scale of Positive and Negative Experience (SPANE; [23]) was used to assess positive (6 items) and negative affect (6 items), including feelings such as joy, contentment, sadness, or anger, during the last week in a 5-point Likert scale (from 1 = very rarely or never to 5 = very often or always). Higher scores represent higher positive affect or higher negative affect. In the present study, Cronbach’s alphas and Omega were, respectively, for positive affect, 0.94 and 0.95, and for negative affect, both 0.86. Sum scores were used for subsequent analyses.

The Spanish version [24] of the 21-item Posttraumatic Growth Inventory (PTGI; [8]) was used to assess PTG in a 6-point Likert scale (from 0 = I did not experience any change to 5 = I experienced a very great change), including different aspects of psychological growth such as relating to others (e.g., “Knowing that I can count on people in times of trouble”), finding new possibilities (e.g., “I established a new path for my life”), personal strength (e.g., “Knowing I can handle difficulties”), spiritual change (e.g., “A better understanding of spiritual matters”), and appreciation of life (e.g., “My priorities about what is important in life”). Higher scores indicate a higher degree of reported positive changes. In the present study, Cronbach’s alpha and Omega were both 0.94 (total score). The sum of its item scores was used for subsequent analyses.

### 2.3. Procedure

This study has a longitudinal design with two data collection times at relevant moments during the pandemic. Time 1 (T1, from 21 March 2020, to 2 April 2020) corresponds approximately to the second week of the lockdown in Spain, and Time 2 (T2, from 24 April 2021, to 18 May 2021) is 13 months after the announcement of the lockdown in Spain. To collect data, we used a snowball sampling method. An online survey was sent by email, telephone messaging services (WhatsApp, Signal), and social networks (Twitter) to colleagues, acquaintances, friends, and family who were asked to spread it. The study was conducted in accordance with the ethical principles outlined in the Declaration of Helsinki. All participants were fully informed of their rights as research participants, the assurance of their anonymity, the purpose of the research, the intended use of their data, and the absence of any associated risks. Participation was entirely voluntary and informed consent was obtained from all participants. Due to the urgent launch of the study during the first week of the COVID-19 lockdown—an unprecedented and unforeseen situation—we were unable to complete the Institutional Review Board (IRB) approval process in advance. However, the study received the Institutional Review Board (IRB) approval after we conducted the study (code 27_25_COVID). At the end of the questionnaire at T1, participants were asked whether they could be contacted in the future. Participants who agreed wrote their email in a blank space and were contacted again via email to answer the questionnaire at T2. Participants completed measures of sociodemographic data (T1), character strengths (T1), MH (T1 and T2), SWB (i.e., life satisfaction, positive affect, and negative affect; T2), and PTG (T2). Although online data collection has been criticized (e.g., for possible sample biases), empirical evidence shows that data obtained online are comparable to data collected in more conventional ways (e.g., [25]). Data are available at https://osf.io/vuhjr/overview?view_only=fae1621710bd44bfb67d5f73e6387042 (accessed on 17 October 2025).

## 3. Results

Results were organized in two main sections. First, we present the results regarding the model of character as a general factor predicting mental health. Second, we present the results regarding the five strengths factors as predictors.

### 3.1. Character as a General Factor Predicting Mental Health

#### 3.1.1. Model Specification

Structural equation modeling (SEM) was used to examine the associations between character as a general factor and mental health at post-test, as well as the potential mediating role of multiple well-being indicators. SEM was selected because it allows for the simultaneous estimation of direct and indirect associations while accounting for correlations among predictors.

For the purposes of the structural analyses and to ensure a parsimonious representation of the constructs involved, a composite variable termed character was defined. In order to do so, a confirmatory factor analysis was conducted to examine whether the five character strength dimensions (fortitude strengths, goodness strengths, intellectual strengths, interpersonal strengths, and strengths of restraint) loaded on a single latent factor. The one-factor model showed good incremental fit (CFI = 0.95), although parsimony-based indices were more modest (TLI = 0.90; RMSEA = 0.14). Given the low degrees of freedom of the model, the RMSEA was interpreted with caution. All five dimensions loaded significantly on the latent factor (λs = 0.55–0.84), supporting the use of a composite character score in subsequent analyses. Therefore, character was operationalized as an observed composite variable computed as the mean of these five strengths factors, all of which were assessed on the same continuous scale. Treating character as an observed variable allowed for a parsimonious representation of the construct while preserving its theoretical scope, which was particularly appropriate given the sample size.

**Figure 1:**
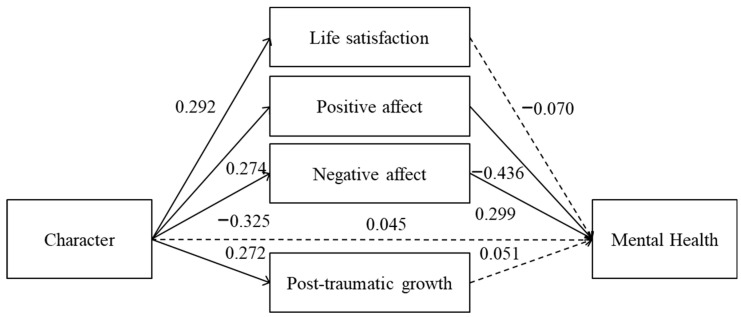
Structural model of the associations between character and mental health at post-test through different well-being indicators. Note. Standardized coefficients (β) are shown. Solid arrows represent significant paths, and dotted arrows represent non-significant paths. Covariates and error terms are omitted for clarity. Lower MH scores indicate better mental health.

The specified model (see Figure 1) included character as the main predictor, mental health at post-test as the outcome variable, and four proposed mediators assessed at post-test: satisfaction with life, positive affect, negative affect, and post-traumatic growth. Direct paths were specified from character to each mediator and to mental health at post-test, as well as from each mediator to mental health at post-test. To control for potential confounding effects, baseline mental health, age, gender, and educational level were included as covariates, each with a direct path to mental health at post-test. All exogenous variables were allowed to covary.

Residual error terms were specified for all endogenous variables. Given the conceptual overlap among the mediating variables and their assessment at the same time point, residual covariances were allowed among satisfaction with life, positive affect, and negative affect. In contrast, residual covariances involving post-traumatic growth were not specified in order to enhance model parsimony and reflect its conceptual distinctiveness from hedonic well-being indicators.

#### 3.1.2. Estimation and Model Evaluation

The model was estimated using maximum likelihood estimation in AMOS 23. Model fit was evaluated using multiple indices, including the chi-square statistic (χ^2^), the chi-square to degrees of freedom ratio (χ^2^/df), the Comparative Fit Index (CFI), the Tucker–Lewis Index (TLI), and the Root Mean Square Error of Approximation (RMSEA). This combination of indices was selected to provide a balanced assessment of absolute fit, incremental fit, and parsimony.

Unstandardized regression coefficients (b) were used for statistical inference and are reported in the text, whereas standardized coefficients (β) are presented in the graphical representation of the model (see Figure 1).

#### 3.1.3. Model Fit

A path model was tested to examine whether the association between character and mental health at post-test was mediated by satisfaction with life, positive affect, negative affect, and post-traumatic growth (see Figure 1), while controlling for baseline general health, age, gender, and educational level. The proposed structural model was evaluated using multiple fit indices, given the well-known sensitivity of individual indices to sample size and model complexity. The chi-square test was statistically significant, χ^2^(19) = 55.66, *p* < 0.001; however, the χ^2^/df ratio (2.93) fell within the range typically considered indicative of acceptable model fit. In addition, the Comparative Fit Index (CFI = 0.91) exceeded the conventional threshold of 0.90, suggesting that the proposed model represented a substantial improvement over the independence model.

Other fit indices indicated more modest levels of parsimony. Specifically, the Tucker–Lewis Index (TLI = 0.77) and the Root Mean Square Error of Approximation (RMSEA = 0.12) fell below commonly recommended cutoffs. However, both indices are known to be particularly sensitive to model complexity, small sample sizes, and a limited number of degrees of freedom. Taken together, these considerations suggest that the model provides an acceptable, albeit not optimal, representation of the data, and that the observed limitations in parsimony-related indices should be interpreted with caution.

#### 3.1.4. Effects of Character on the Mediators

Character showed significant associations with all proposed mediators (see Figure 1). Specifically, higher levels of character predicted higher satisfaction with life (b = 1.18, SE = 0.32, *p* < 0.001), higher positive affect (b = 1.30, SE = 0.38, *p* < 0.001), lower negative affect (b = −1.73, SE = 0.42, *p* < 0.001), and higher post-traumatic growth (b = 0.22, SE = 0.06, *p* < 0.001). These results indicate that character is robustly related to all mediators at post-test.

#### 3.1.5. Effects of Mediators on Mental Health at Post-Test

When all mediators were entered simultaneously into the model, only the affective variables were significantly associated with mental health at post-test. Positive affect was negatively associated with mental health scores (b = −0.57, SE = 0.10, *p* < 0.001), indicating better mental health at higher levels of positive affect. Negative affect was positively associated with mental health scores (b = 0.35, SE = 0.08, *p* < 0.001), indicating poorer mental health at higher levels of negative affect. Considering the mediation effects as a whole and based on the product of path coefficients, the indirect association between character and mental health was larger through positive affect (b ≈ −0.74) than through negative affect (b ≈ −0.61).

In contrast, satisfaction with life (b = −0.11, SE = 0.11, *p* = 0.316) and post-traumatic growth (b = 0.39, SE = 0.41, *p* = 0.340) were not significantly related to mental health at post-test once affective variables were controlled.

#### 3.1.6. Direct Effect of Character on Mental Health at Post-Test

The direct path from character to mental health at post-test was not significant (b = 0.28, SE = 0.36, *p* = 0.447). This suggests that the relationship between character and mental health was fully accounted for by the mediating variables included in the model.

Taken together, the results support a pattern of full mediation, whereby the association between character and mental health at post-test operates primarily through affective processes. Although character was significantly related to all proposed mediators, only positive affect and negative affect were directly associated with mental health, indicating that the affective component of well-being plays a central role in linking character strengths to mental health in the longitudinal context examined.

### 3.2. The Five Specific Character Strengths Factors Predicting Mental Health

In addition to the general model tested above, we conducted a series of parallel mediation models with each of the five character strength factors as predictors (measured at T1), satisfaction with life, positive affect, negative affect, and post-traumatic growth as mediators (measured at T2), and MH as outcome (measured at T2). These complementary analyses allowed us to examine in more detail whether specific strength factors have different effects on well-being and mental health.

#### 3.2.1. Assumption Testing

The statistical assumptions relevant to the mediation model (i.e., assumptions of no univariate outliers, no multivariate outliers, normality of residuals, linearity of residuals, homoskedasticity of residuals, no multicollinearity, and independence of errors) were met (see Appendix A).

#### 3.2.2. Descriptive Statistics and Intercorrelations Among Character Strengths Factors, Indicators of Well-Being and Mental Health

Table 3 displays the descriptive statistics and intercorrelations among the variables of the study.

#### 3.2.3. Mediation Analyses

Five parallel mediations models with bootstrapped 95% confidence intervals (5000 iterations) were examined using PROCESS macro 4.2 [26] (Model 4), for SPSS 29. Each analysis examined a model that included one of the five character strengths factors as the predictor, MH at T2 as the outcome, life satisfaction, positive affect, negative affect, and PTG at T2 as the mediators, and gender, age, education, and MH at T1 as covariates. Mediation was determined when the confidence intervals of the indirect effects did not include zero. Next, we describe the five parallel mediation analyses for each of the five character strengths factors. Unstandardized coefficients are reported in the text while standardized coefficients are reported in the figures.

##### Fortitude Strengths

**Figure 2:**
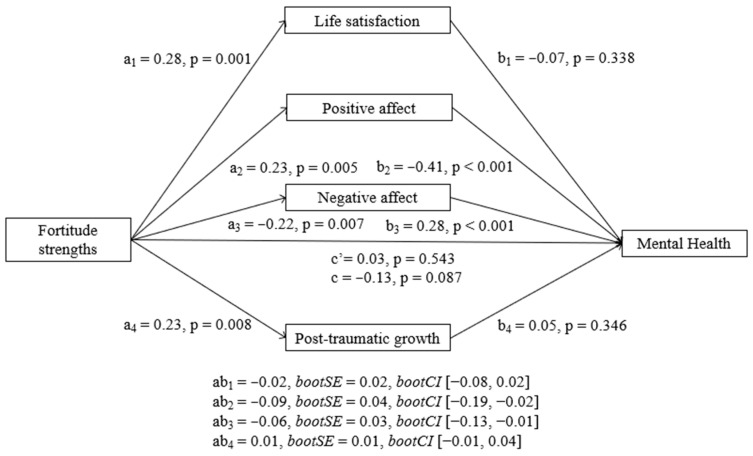
Parallel mediation model with standardized effects of the relationship between fortitude strengths and mental health mediated by life satisfaction, positive affect, negative affect, and post-traumatic growth. Lower MH scores indicate better mental health.

Results from the parallel mediation analysis indicated that fortitude strengths are indirectly associated with MH at T2 through its relationship with both positive and negative affect. Figure 2 shows the standardized pathways of the model with the fortitude strengths factor as the predictor. Covariates in the model are not represented in the figure for simplicity. First, as can be seen in Figure 2, the total effect of fortitude strengths on MH at T2 was negative and not significant, although there was a tendency (b = −0.61, SE = 0.35, t = −1.71, *p* = 0.087). The direct effect of fortitude strengths on MH at T2 was not significant (b = 0.16, SE = 0.27, t = 0.60, *p* = 0.543), indicating that there is not a direct relationship between fortitude and MH at T2 when considering the mediators. However, positive affect (b = −0.45, BootSE = 0.21, BootLLCI = −0.93, BootULCI = −0.10) and negative affect (b = −0.29, BootSE = 0.14, BootLLCI = −0.62, BootULCI = −0.05) had significant indirect effects (a*b). According to the signs of the coefficients reported in Figure 2 (a2, b2, a3, b3), fortitude strengths predicted a better MH at T2 through higher positive affect and lower negative affect. Life satisfaction and PTG did not show significant indirect effects individually. Additionally, pairwise contrasts comparing the strength of the indirect effects [27] revealed that the indirect effects of positive affect and negative affect were significantly different from the indirect effect of PTG. There were no other significant differences in the strength of the indirect effects. Pairwise contrasts comparing the strength of the indirect effects have been included in Appendix B.

##### Goodness Strengths

**Figure 3:**
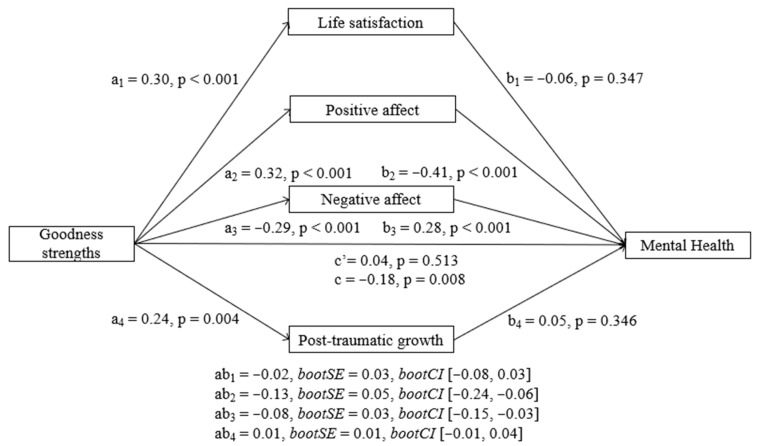
Parallel mediation model with standardized effects of the relationship between goodness strengths and mental health mediated by life satisfaction, positive affect, negative affect, and post-traumatic growth. Lower MH scores indicate better mental health.

Results showed that goodness strengths had a significant negative total effect (b = −1.00, SE = 0.37, t = −2.67, *p* = 0.008) and a significant indirect effect on MH at T2 through its relationship with both positive and negative affect (see Figure 3). The direct effect of goodness strengths on MH at T2 was not significant (b = 0.20, SE = 0.30, t = 0.65, *p* = 0.513). However, positive affect (b = −0.73, BootSE = 0.26, BootLLCI = −1.30, BootULCI = −0.30) and negative affect (b = −0.44, BootSE = 0.16, BootLLCI = −0.80, BootULCI = −0.17) had significant indirect effects. As can be seen in Figure 2, goodness strengths also predicted a better MH at T2 through higher positive affect and lower negative affect. Life satisfaction and PTG did not show significant indirect effects individually. In addition, pairwise contrasts comparing the strength of the indirect effects showed that the indirect effect of positive affect was significantly different from the indirect effects of both life satisfaction and PTG and that the indirect effect of negative affect was significantly different from the indirect effect of PTG.

##### Intellectual Strengths

**Figure 4:**
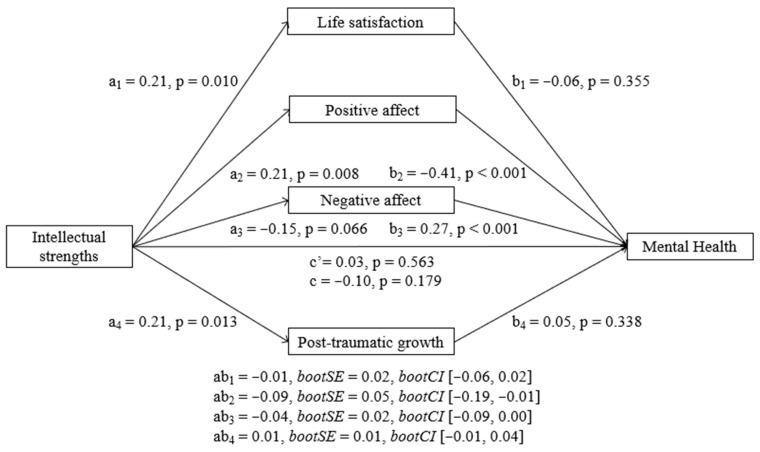
Parallel mediation model with standardized effects of the relationship between intellectual strengths and mental health mediated by life satisfaction, positive affect, negative affect, and post-traumatic growth. Lower MH scores indicate better mental health.

Results showed that intellectual strengths did not have a significant total effect (b = −0.55, SE = 0.41, t = −1.34, *p* = 0.179) nor a direct effect (b = 0.18, SE = 0.31, t = 0.57, *p* = 0.563). However, intellectual strengths had a significant indirect effect on MH at T2 through its relationship with positive affect (b = −0.49, BootSE = 0.26, BootLLCI = −1.08, BootULCI = −0.08), so higher intellectual strengths were associated with higher positive affect and higher positive affect with better mental health (see Figure 4). Negative affect, life satisfaction and PTG did not show any significant indirect effects individually. In addition, pairwise contrasts comparing the strength of the indirect effects showed that the indirect effect of positive affect was significantly different from the indirect effects of PTG and that the indirect effect of negative affect was significantly different from the indirect effect of PTG.

##### Restraint Strengths

**Figure 5:**
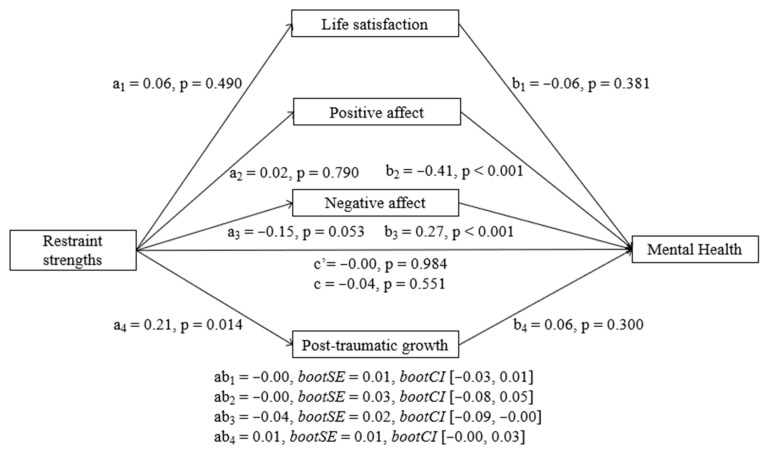
Parallel mediation model with standardized effects of the relationship between restraint strengths and mental health mediated by life satisfaction, positive affect, negative affect, and post-traumatic growth. Lower MH scores indicate better mental health.

Results showed that restraint strengths did not have a significant total effect (b = −0.20, SE = 0.33, t = −0.59, *p* = 0.551) nor a direct effect (b = −0.00, SE = 0.25, t = −0.01, *p* = 0.984). Strengths of restraint only predicted PTG significantly and there was a tendency in relation to negative affect, but these strengths did not predict positive affect nor life satisfaction. However, restraint strengths had a significant indirect effect on MH at T2 through its relationship with negative affect (b = −0.20, BootSE = 0.12, BootLLCI = −0.47, BootULCI = −0.00), so higher restraint strengths were associated with lower negative affect and lower negative affect with better mental health (Figure 5). Positive affect, life satisfaction, and PTG did not show any significant indirect effects. In addition, pairwise contrasts comparing the strength of the indirect effects showed that the indirect effect of negative affect was significantly different from the indirect effect of PTG.

##### Interpersonal Strengths

**Figure 6:**
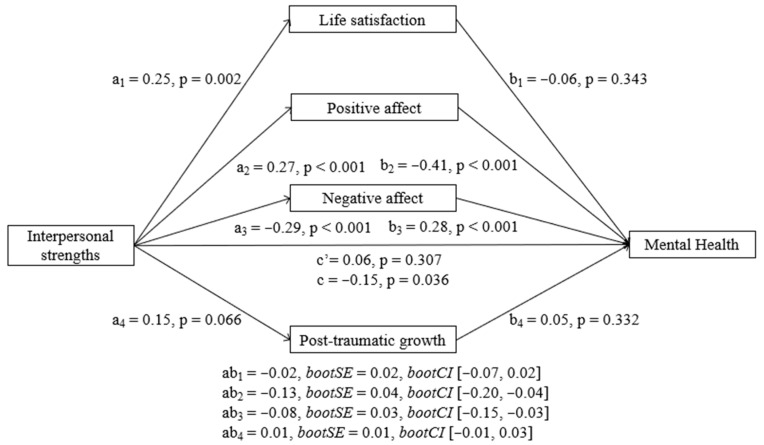
Parallel mediation model with standardized effects of the relationship between interpersonal strengths and mental health mediated by life satisfaction, positive affect, negative affect, and post-traumatic growth. Lower MH scores indicate better mental health.

Results showed that interpersonal strengths had a significant negative total effect (b = −0.71, SE = 0.34, t = −2.11, *p* = 0.036) and a significant indirect effect on MH at T2 through its relationship with both positive (b = −0.55, BootSE = 0.19, BootLLCI = −0.98, BootULCI = −0.22) and negative affect (b = −0.39, BootSE = 0.14, BootLLCI = −0.71, BootULCI = −0.16). The direct effect of interpersonal strengths on MH at T2 was not significant (b = 0.27, SE = 0.26, t = 1.02, *p* = 0.307). As Figure 6 shows, interpersonal strengths predicted a better MH at T2 through higher positive affect and lower negative affect. Life satisfaction and PTG did not show significant indirect effects individually. In addition, pairwise contrasts comparing the strength of the indirect effects showed that the indirect effect of positive affect was significantly different from the indirect effects of both life satisfaction and PTG and that the indirect effect of negative affect was significantly different from the indirect effect of PTG.

## 4. Discussion

This study explored whether character strengths predict better mental health over a period of 13 months during the COVID-19 pandemic. Additionally, it tested different routes that might explain this relationship. In particular, four different well-being-related variables were explored as mediators simultaneously: life satisfaction, positive and negative affect, as reflecting affective, cognitive, and hedonic aspects of well-being, and PTG, as reflecting positive growth following adversity. We first examined a model with one general character factor as a predictor of MH. Then, we conducted a series of more detailed mediational models with each of the five character strengths factors as predictors of MH.

Regarding the use of character as a single global factor, various authors have suggested that the different character strengths seem to contribute jointly to a “good character” [10,28]. Our data supported that idea and, moreover, showed that the association between character, as a single general factor, and MH at T2 operates primarily through affective processes, especially through positive affect. Character predicted all four mediators, but only the affective components of well-being predicted MH. This model, which is parsimonious and easy to interpret, helps us synthesize previous evidence regarding the relationship between character strengths, different conceptualizations of well-being, and MH in adverse situations. Moreover, it highlights the special role of positive affect as the most important route linking character and MH.

When going into detail and analyzing the five character strengths separately, we observed that not all character strength factors operated equally, which supports the use of the additional mediational analyses. Intellectual strengths did not predict negative affect, strengths of restraint only predicted PTG, and interpersonal strengths did not predict PTG. Nonetheless, all character strength factors predicted MH indirectly through the affective component of well-being, i.e., through higher positive and/or lower negative affect. The positive affect pathway showed the strongest effect sizes, except in the case of strengths of restraint, where only the negative affect pathway was statistically significant. The life satisfaction and PTG pathways were not statistically significant, mainly because life satisfaction and PTG were not related to MH.

Since this study is an extension of a previous investigation that explored the relationship between character strengths and well-being and MH over one month during the initial stages of the pandemic [9], we can compare now which character strengths factors remained as significant predictors of well-being and MH in the long run. In comparison with the first study [9], where all character strengths predicted MH one month later, results showed that only goodness (i.e., love, kindness, gratitude, forgiveness, and integrity) and interpersonal strengths (i.e., humor, citizenship, and social intelligence) had a significant total effect on mental health 13 months later. Thus, our expectations were partially met. Although these comparisons should be interpreted carefully, this is an interesting finding since fortitude strengths yielded the highest correlations with MH and SWB in the previous study. According to these results, it seems that in the context of challenging situations such as the pandemic, character strengths more related to goodness and interpersonal relationships might be especially important for MH in the long run. Like the previous study, all character strength factors, except strengths of restraint, predicted higher positive affect. However, in the previous study, only intellectual and interpersonal strengths predicted higher life satisfaction, while in the present study, all character strengths, except strengths of restraint, predicted life satisfaction. Regarding negative affect, in the previous study, fortitude strengths, interpersonal strengths, and strengths of restraint, predicted lower negative affect, while in the present study, fortitude, interpersonal, and goodness did (although the effects of strengths of restraint and intellectual strengths were very close to the significance level). Finally, all character strengths except interpersonal strengths (although there was a tendency) predicted PTG, adding more evidence to the relationship between character strengths and PTG in the context of the pandemic. Goodness and fortitude strengths were, indeed, the best predictors of PTG, which is in line with previous studies (e.g., [10,11]).

Overall, these results are in line with previous studies (e.g., [9,10,11,29,30]) and highlight the relevant role of character strengths for well-being and MH. Among all character strengths, goodness strengths, interpersonal strengths, and fortitude strengths were generally the best predictors. These strengths broadly overlap with the virtues of humanity (interpersonal strengths that involve ‘‘tending and befriending’’ others) and transcendence (strengths that allow us to connect with something outside ourselves and make sense of the larger universe, moving beyond the physical world and the concrete), according to Peterson and Seligman’s model [5]. Thus, these results suggest that, especially in the long term, character strengths that particularly relate to humanity and transcendence might be especially beneficial when facing adverse situations such as the COVID-19 pandemic. Maybe these character strengths have helped individuals nourish a sense of connection or closeness with the transcendent, oneself, and humanity, bringing meaning and purpose in those difficult times and fostering a better general psychological functioning. Moreover, character strengths might nourish a better mental health through a greater experience of positive affect.

This study has several limitations and results should be interpreted carefully. Firstly, we used a convenience sample, which is not representative of the general Spanish population. Secondly, given that we analyzed the potential protective role of character strengths in a Spanish sample, it could be argued that the results observed in the present study cannot be generalized to other countries. While this is true, it should be noted that the results observed in this study are relatively aligned with those found in other countries such as Italy, Germany, Switzerland, and Austria (e.g., [10,11]). Thirdly, given that this is a longitudinal study, we cannot infer any causality in a strict sense. Also, it must be noted that the mediators and MH were assessed at the same time (T2). However, the study suggests that character strengths relate longitudinally to better mental health during the pandemic. Fourthly, all variables were self-reported and collected online, thus shared method variance may inflate the observed associations. Accordingly, the indirect effects through affect may reflect stable individual differences in emotional style. Also, in the Character Strengths Rating Form, all items are positively worded, which may inflate inter-item correlations by acquiescence bias. Regarding the Post-traumatic Growth Inventory, its response format implicitly assumes that change following adversity is positive. Participants can only report stability or improvement across domains, with no option to indicate negative change. This might lead to a conflation of positive reinterpretation with genuine growth and may inflate associations with adaptive traits or positive affect [31,32]. Restricted variance may bias the interpretation of mediation models. In order to overcome these limitations, future studies should use more representative samples, assess mediators and outcome variables at different times, and use more objective measures that complement self-report measures.

## 5. Conclusions

This study offers original evidence regarding the relationship between character strengths, hedonic well-being, post-traumatic growth, and mental health over 13 months during the COVID pandemic. We have demonstrated that a general single factor of character predicts later positive affect, negative affect, life satisfaction, post-traumatic growth, and mental health in challenging circumstances, and that it is mainly through the affective component of well-being, especially through higher positive affect, that character strengths relate longitudinally to mental health. In fact, life satisfaction (i.e., the cognitive component of subjective well-being) and post-traumatic growth were not related to mental health. When looking at the five character strengths factors, although the majority predicted higher well-being and better mental health over time, goodness and interpersonal and fortitude strengths yielded the strongest effects. Future studies would be beneficial to replicate these findings in other adverse contexts besides a pandemic and with more representative and larger samples to be able to draw more robust conclusions.

## Figures and Tables

**Table 1:** Virtues and Character Strengths.

Virtue	General Description	Associated Strengths
Wisdom and Knowledge	Cognitive strengths that support the acquisition, integration, and practical use of knowledge.	Creativity: generating original and useful ideas or approaches. Curiosity: showing an active interest in new experiences and learning opportunities. Open-mindedness: considering multiple viewpoints before making judgments or decisions. Love of learning: finding enjoyment in mastering new skills or areas of knowledge. Perspective: offering sound advice and seeing issues with depth and understanding.
Courage	Emotional strengths that enable individuals to pursue goals with determination despite fear, difficulty, or opposition.	Bravery: confronting challenges or threats without avoidance. Perseverance: staying committed and completing what one has started. Integrity or authenticity: acting and speaking truthfully, in alignment with personal values. Vitality or zest: approaching life with enthusiasm and energy.
Humanity	Interpersonal strengths that foster caring, connection, and mutual support.	Love: valuing close, warm relationships with others. Kindness: performing helpful or compassionate actions toward others. Social intelligence: understanding the feelings and motives of oneself and others.
Justice	Civic strengths that sustain fairness, cooperation, and collective well-being.	Teamwork or citizenship: contributing constructively as a member of a group or community. Fairness: treating people impartially and with a sense of justice. Leadership: guiding and motivating others to achieve shared goals.
Temperance	Strengths that help regulate impulses and guard against excess.	Forgiveness: letting go of resentment toward those who have caused harm. Humility or modesty: allowing achievements to speak for themselves and recognizing one’s limitations. Prudence: thinking carefully before acting or speaking to avoid later regret. Self-regulation: managing emotions and behaviors in balanced ways.
Transcendence	Strengths that connect individuals with a broader sense of meaning and purpose in life.	Appreciation of beauty and excellence: noticing and valuing beauty, moral excellence, and skill in the world. Gratitude: recognizing and being thankful for positive events and experiences. Hope: maintaining optimism and working toward favorable outcomes. Humor: enjoying laughter and spreading joy to others. Spirituality: holding beliefs or values that provide a sense of higher meaning or purpose.

Note: Adapted from Martínez-Martí and Ruch [4].

**Table 2:** Character strengths factors reported in Martínez-Martí et al. [9].

(1) Fortitude strengths: spirituality, bravery, persistence, hope, leadership, and zest.
(2) Goodness strengths: kindness, love, gratitude, forgiveness, and integrity.
(3) Intellectual strengths: curiosity, love of learning, open-mindedness, creativity, perspective, and appreciation of beauty and excellence.
(4) Strengths of restraint: prudence, self-regulation, humility, and fairness.
(5) Interpersonal strengths: humor, citizenship, and social intelligence.

**Table 3:** Descriptive statistics and intercorrelations among variables (N = 146).

	1	2	3	4	5	6	7	8	9	10	11	12	13
1. FS	-												
2. GS	0.56 **	-											
3. IntelS	0.55 **	0.43 **	-										
4. RS	0.44 **	0.60 **	0.24 **	-									
5. InterS	0.54 **	0.68 **	0.44 **	0.51 **	-								
6. MH T1	−0.24 **	0.00	−0.06	−0.06	−0.12	-							
7. MH T2	−0.25 **	−0.18 *	−0.12	−0.08	−0.22 **	0.56 **	-						
8. LS	0.31 **	0.29 **	0.21 *	0.07	0.27 **	−0.27 **	−0.52 **	-					
9. PA	0.26 **	0.29 **	0.22 **	0.02	0.30 **	−0.40 **	−0.70 **	0.67 **	-				
10. NA	−0.30 **	−0.29 **	−0.16	−0.18 *	−0.34 **	0.40 **	0.67 **	−0.49 **	−0.64 **	-			
11. PTG	0.24 **	0.25 **	0.21 *	0.22 **	0.15	−0.04	−0.10	0.22 **	0.26 **	−0.11	-		
12. Gender	0.04	0.05	−0.01	0.03	−0.08	0.29 **	0.24 **	−0.01	−0.17 *	0.16	0.13	-	
13. Age	0.23 **	0.16 *	0.12	0.19 *	0.10	−0.10	−0.11	−0.02	−0.09	−0.10	0.03	−0.09	-
14. Education	−0.02	−0.02	0.25 **	−0.00	−0.07	−0.01	0.04	−0.00	0.07	0.04	−0.00	−0.06	0.01
M	6.19	7.29	6.92	6.44	6.98	13.12	13.95	17.78	21.36	15.95	1.87		
SD	1.30	1.17	1.12	1.34	1.32	5.55	6.34	3.92	4.59	5.18	0.77		

Note. * *p* < 0.05; ** *p* < 0.01. (two tailed). Gender: 1 = male, 2 = female. The correlations presented are Pearson correlations. FS = Fortitude strengths. GS = Goodness strengths. IntelS = Intellectual strengths. RS = Restraint strengths. InterS = Interpersonal strengths. MH = mental health. PTG = post-traumatic growth. LS = Life satisfaction. PA = Positive affect. NA = Negative affect. Lower MH scores indicate better mental health.

## Data Availability

Data are available at: https://osf.io/vuhjr/overview?view_only=fae1621710bd44bfb67d5f73e6387042 (accessed on 17 October 2025).

## Appendix A

All data was analyzed with SPSS v29. All the assumptions were tested for a multiple regression model with five predictors (a character strengths factor, life satisfaction, positive affect, negative affect, PTG) predicting mental health. While one univariate outlier was identified in MH at T1 and strengths of restraint scores, two univariate outliers in goodness, intellectual and interpersonal strengths, and PTG scores, and three multivariate outliers were identified with Mahalanobis distance > 20.52; none of these cases had Cook’s distance > 1, indicating they were not influencing the model, and thus were retained. The Shapiro–Wilk statistics for MH at T1, MH at T2, positive affect, goodness, interpersonal, and PTG did not meet normality (*p* < 0.001); however, those for age, satisfaction with life, negative affect, fortitude, intellectual, and restraint strengths had a normal distribution. Skewness and kurtosis were within the range of +/− 2 for all variables, satisfying univariate normality. Linearity of residuals and homoscedasticity of residuals were assessed through inspection of the scatterplot of the standardized observed residuals against standardized predicted values. The data points appeared scattered randomly across the horizontal axis with no obvious curve, indicating linearity of residuals. The scatterplot showed the points were spread out and showed no clear pattern, indicating homoscedasticity. Normality of residuals were assessed through the inspection of the normal P–P plot regression of standardized residuals The residual data points showed a slight deviation from the diagonal line which indicates nonnormality. Independence of errors was indicated by the Durbin–Watson statistic of 2.066, which is between the acceptable range of one to three. Multicollinearity was examined by inspecting bivariate correlations, revealing all correlations to be <0.85 and all Tolerance and Variance Inflation Factors were >0.10 and <10, respectively, meeting this assumption.

## Appendix B. Pairwise Contrasts Comparing the Strength of the Indirect Effects

**Table A1:** Pairwise Contrasts Comparing the Strength of the Indirect Effects.

	Completely Standardized Indirect Effect(s) of X on Y				
	Effect	BootSE	BootLLCI	BootULCI	
Fortitude strengths	(C1)	0.0743	0.0558	−0.0209	0.1988
	(C2)	0.0423	0.0439	−0.0427	0.1360
	(C3)	−0.0304	0.0298	−0.1024	0.0185
	(C4)	−0.0320	0.0409	−0.1202	0.0430
	(C5)	−0.1047	0.0484	−0.2174	−0.0252
	(C6)	−0.0727	0.0339	−0.1493	−0.0177
Goodness strengths	(C1)	0.1133	0.0639	0.0023	0.2516
	(C2)	0.0608	0.0463	−0.0264	0.1562
	(C3)	−0.0317	0.0293	−0.0960	0.0201
	(C4)	−0.0525	0.0444	−0.1486	0.0269
	(C5)	−0.1450	0.0508	−0.2596	−0.0618
	(C6)	−0.0925	0.0323	−0.1630	−0.0374
Intellectual strengths	(C1)	0.0723	0.0553	−0.0174	0.1970
	(C2)	0.0265	0.0357	−0.0410	0.1014
	(C3)	−0.0248	0.0245	−0.0822	0.0132
	(C4)	−0.0458	0.0400	−0.1351	0.0224
	(C5)	−0.0971	0.0494	−0.2123	−0.0190
	(C6)	−0.0512	0.0276	−0.1121	−0.0041
Restraint strengths	(C1)	0.0050	0.0328	−0.0554	0.0748
	(C2)	0.0376	0.0275	−0.0123	0.0968
	(C3)	−0.0152	0.0164	−0.0556	0.0085
	(C4)	0.0326	0.0284	−0.0241	0.0882
	(C5)	−0.0202	0.0367	−0.0996	0.0474
	(C6)	−0.0528	0.0275	−0.1134	−0.0051
Interpersonal strengths	(C1)	0.0968	0.0530	0.0025	0.2139
	(C2)	0.0653	0.0407	−0.0122	0.1492
	(C3)	−0.0241	0.0245	−0.0779	0.0195
	(C4)	−0.0315	0.0445	−0.1229	0.0515
	(C5)	−0.1209	0.0442	−0.2178	−0.0454
	(C6)	−0.0894	0.0312	−0.1568	−0.0367

Note. Specific indirect effect contrast definition(s): (C1) = Life satisfaction minus Positive affect; (C2) = Life satisfaction minus Negative affect; (C3) = Life satisfaction minus PTG; (C4) = Positive affect minus Negative affect; (C5) = Positive affect minus PTG; (C6) = Negative affect minus PTG.
